# Geographic Variation in Venom Allelic Composition and Diets of the Widespread Predatory Marine Gastropod *Conus ebraeus*

**DOI:** 10.1371/journal.pone.0006245

**Published:** 2009-07-16

**Authors:** Thomas F. Duda, Dan Chang, Brittany D. Lewis, Taehwan Lee

**Affiliations:** 1 Department of Ecology and Evolutionary Biology and Museum of Zoology, University of Michigan, Ann Arbor, Michigan, United States of America; 2 Smithsonian Tropical Research Institute, Balboa, Ancón, Republic of Panama; American Museum of Natural History, United States of America

## Abstract

**Background:**

Members of the predatory gastropod genus *Conus* use a venom comprised of a cocktail of peptide neurotoxins, termed conotoxins or conopeptides, to paralyze prey and conotoxin gene family members diversify via strong positive selection. Because *Conus* venoms are used primarily to subdue prey, the evolution of venoms is likely affected by predator-prey interactions.

**Methodology/Principal Findings:**

To identify the selective forces that drive the differentiation of venoms within species of *Conus*, we examined the distribution of alleles of a polymorphic O-superfamily conotoxin locus of *Conus ebraeus* at Okinawa, Guam and Hawaii. Previous analyses of mitochondrial cytochrome oxidase I gene sequences suggest that populations of *C. ebraeus*, a worm-eating *Conus*, are not structured genetically in the western and central Pacific. Nonetheless, because the sample size from Guam was relatively low, we obtained additional data from this location and reexamined patterns of genetic variation at the mitochondrial gene at Okinawa, Guam and Hawaii. We also utilized a DNA-based approach to identify prey items of individuals of *C. ebraeus* from Guam and compared this information to published data on diets at Okinawa and Hawaii. Our results show that conotoxin allelic frequencies differ significantly among all three locations, with strongest differentiation at Hawaii. We also confirm previous inferences that *C. ebraeus* exhibits no genetic differentiation between Okinawa, Guam and Hawaii at the mitochondrial locus. Finally, DNA-based analyses show that eunicid polychaetes comprise the majority of the prey items of *C. ebraeus* at Guam; while this results compares well with observed diet of this species at Okinawa, *C. ebraeus* preys predominantly on nereid polychaetes at Hawaii.

**Conclusions/Significance:**

These results imply that strong selection pressures affect conotoxin allelic frequencies. Based on the dietary information, the selection may derive from geographic variation in dietary specialization and local coevolutionary arms races between *Conus* and their prey.

## Introduction

Members of the predatory marine gastropod genus *Conus* utilize a complex venom comprised of numerous neurotoxic peptides, termed conotoxins, to paralyze prey [1]. Conotoxins are encoded by members of large gene families and target a variety of different ion channels and cell receptors in prey [1]. Previous analyses of conotoxin gene family evolution reveal that conotoxins are subject to very strong positive selection [2]–[6]. Because venoms are used primarily to subdue prey, the selective forces responsible for the evolution of venom components are likely associated with predator-prey interactions. In particular, an arms race may occur between the conotoxins of *Conus* and the neuronal receptors and ion channels of prey, or *Conus* venoms may evolve to track changes in dietary specializations [4]. Although it is difficult to specifically test these hypotheses, information on the ecological and genetic correlates of variation in venom composition within species will enhance our understanding of the forces driving *Conus* venom evolution.

Snakes [7]–[10] and scorpions [11] show geographic variation in venom composition and these differences are potentially associated with differences in diets among populations of snakes, although this relationship is controversial [8], [9], [12], [13]. For example, Daltry *et al.* [9] reported that venom composition and diets of populations of a Malayan pitviper are correlated, but it is unclear what factors were responsible for the differences in venom composition among populations because venoms were characterized based on differences in isoelectric focusing patterns of whole venoms. Results from other venomous snakes suggest that coevolutionary arms races drive the evolution of venoms [14]. Nonetheless, very few studies have directly investigated intraspecific patterns of variation of genes involved with envenomation.

Recently, Duda and Lee [15] observed significant differences in allelic frequencies of two conotoxin loci of *Conus miliaris* at Easter Island, a population that has undergone ecological release at this isolated location and exhibits a broader dietary breadth than at other locations in the Indo-West Pacific [16]. These results suggest that strong selection pressures associated with dietary specialization affect venom composition of *Conus* and similar phenomena may affect the evolution of venoms of other taxa.

*Conus ebraeus* is one of the most widely distributed members of its genus and occurs in shallow water, tropical regions throughout the Indo-West and eastern Pacific, from the Red Sea to the shores of the Americas [17]. Duda and Palumbi [5] detected a polymorphic O-superfamily conotoxin locus of *C. ebraeus* (conotoxin locus *E1*) and identified two alleles at this locus. These alleles differ at nine nonsynonymous sites that are responsible for 7 amino acid substitutions within the 28 amino acids of the mature conotoxin peptides [5].

To identify the selective forces that drive the differentiation of venoms within species of *Conus* we examined intraspecific patterns of variation at the *E1* locus of *C. ebraeus* at Okinawa, Guam and Hawaii. In particular, we genotyped individuals from Okinawa and Guam in the western Pacific and Hawaii in the central Pacific and compared allelic frequencies among locations. Previous phylogeographic analyses of mitochondrial cytochrome oxidase I (*COI*) sequences of populations of *C. ebraeus* in the Indo-West Pacific show no evidence of population genetic structure among samples from these regions [18], a pattern that is not necessarily unexpected given the potential for wide dispersal in this species due to a three to four week planktonic larval stage [19]. Nonetheless, because the sample size from Guam was relatively low (*n* = 10), we obtained *COI* sequences from additional individuals from Guam to further scrutinize the apparent lack of genetic differentiation of *C. ebraeus* at these locations. While diets of *C. ebraeus* from Hawaii and Okinawa have been described, information from Guam was not previously known. Thus to determine if differences in dietary specialization among samples of *C. ebraeus* from these three locations are associated with any observed differences in allelic frequencies at conotoxin locus *E1*, we described diets of individuals from Guam and compared these data to published information on diets of *C. ebraeus* from Okinawa [20] and Hawaii [21].

## Methods

### Specimen and fecal sample collections

We obtained specimens of *C. ebraeus* from Sesoko Island, Okinawa; Pago Bay, Guam; and Kahe Point, Oahu, Hawaii. These locations are similar in that they consist of shallow subtidal reef habitats. For specimens from Guam, we placed single snails in individual containers (3 ounce plastic cups) with approximately 100 ml of seawater. Containers were examined every 6–12 hours to determine if specimens had defecated. When feces were observed, they were removed with small plastic pipettes and placed in 2 ml centrifuge tubes. As much seawater as possible was then removed from samples and approximately 2 ml of 95% ethanol was added to each vial. Venom ducts of specimens from Guam and Hawaii were dissected from individual snails and stored in RNAlater (Ambion) as per manufacturer's recommendations. Other snail tissues were stored in 70–95% ethanol.

### Conotoxin *E1* allelic sequences and analyses

We prepared cDNA from venom duct mRNA as described previously [4] from 18 individuals of *C. ebraeus* from Hawaii, 29 individuals from Guam and 5 individuals from Okinawa. We extracted genomic DNA (gDNA) using the E.Z.N.A.™ Mollusc DNA Kit (Omega Bio-Tek, Doraville, Georgia, USA) from approximately 25 mg of foot tissue of an additional 13 individuals from Okinawa.

The primers TOX1 (CATCGTCAAGATGAAACTGACGTG) and TOX2 (CACAGGTATGGATGACTCAGG) of Duda and Palumbi [4] were used to amplify alleles of locus *E1* from cDNA. Because O-superfamily conotoxin genes contain a large intron just upstream from the conotoxin coding region, we designed locus-specific primers to amplify locus *E1* from gDNA (preE1: AAACTCCAAGTGGACCAGGGAATG; 3utrE1: GGAAATATCAGGCGCCCCACG).

Amplification products from all but 19 of the individuals from Guam were cloned using a TA cloning kit (Invitrogen). Inserts from at least five positive clones of each product were sequenced, giving greater than 93% probability that both alleles of heterozygotes were detected (*i.e.*, *P* = 1−0.5^(*n*−1)^, where *P* = the probability that both alleles are detected and *n* = the number of inserts sequenced), or until two distinct allelic sequences were identified. Because sequences of a second, apparently monomorphic locus were detected from two individuals from Okinawa and Guam (i.e., locus *E2* from [22]), additional inserts were sequenced from these individuals until at least five *E1* allele sequences were obtained. On average we sequenced 9 inserts per amplification product.

Sequences of *E1* alleles were aligned in Sequencher 4.8 using the contig assembly tool with assembly parameters set to 100% sequence identity to identify sets of identical sequences from multiple individuals. All putative amplification artifacts were removed from subsequent analyses.

We directly sequenced amplification products from cDNA of 19 individuals from Guam as well as from cDNA and gDNA of the 18 individuals from Okinawa (amplifications of the individuals from Okinawa were also cloned as described above; the direct sequencing was conducted to verify results from cloning). The resultant chromatograms of these sequences were examined for the presence of double peaks and genotypes of individuals were determined based on confirmed allele sequences obtained previously from other individuals.

We examined the relationships of alleles and their geographic segregation by constructing a statistical parsimony network [23] with TCS 1.21 [24]. To determine if significant differences in allelic frequencies occur among samples from the three locations, we estimated pairwise F-statistics and conducted an analysis of molecular variance (AMOVA) [25] with Arlequin [26]. The program performed 10,010 permutations to assess the degree to which obtained estimates were different from those obtained from a random assignment of alleles to populations. We used Modeltest 3.7 [27] to determine the most appropriate model of nucleotide substitution and used this model in all relevant calculations in Arlequin.

### COI sequences and analyses

We extracted DNA using the E.Z.N.A.™ Mollusc DNA Kit (Omega Bio-Tek, Doraville, Georgia, USA) from approximately 25 mg of foot tissue of 22 specimens of *C. ebraeus* from Guam. We amplified a region of approximately 644 bases of the mitochondrial COI gene with Folmer primers LCO1490 and HCO2198 [28]. We prepared the products for cycle sequencing by diluting 1∶5 in sterile water. Sequencing was performed in both directions at the University of Michigan DNA Sequencing Core. We analyzed chromatograms with Sequencher 4.8 (Gene Codes Corporation, Ann Arbor, Michigan, USA) and used a text editor to align sequences to published *COI* sequences of 18 individuals of *C. ebraeus* from Okinawa, 10 individuals from Guam and 17 from Hawaii (GenBank Accession numbers EF547559-EF547576, EF547612-EF547628 and EF547602-EF547611).

We constructed a statistical parsimony network [23] of *COI* haplotype sequences with TCS version 1.21 [24]. We calculated pairwise *Φ*_ST_ values and conducted an AMOVA with Arlequin version 2.0 [26]. As above, 10,100 permutations of the data were performed to determine the degree to which obtained estimates were different than those obtained from a random assignment of haplotypes to populations. As in Duda and Lessios [18], we used Tamura-Nei distances [29] in all relevant calculations.

### Identification of prey

In most cases, feces of *C. ebraeus* contain small pieces of undigested prey and *Conus* individuals typically consume a single prey item every other night [21]. Feces of specimens of *C. ebraeus* from Guam were examined with a compound microscope. Based on characteristics of setae, acicula and jaw parts present in feces we tentatively identified prey as eunicid (Order Eunicida, Family Eunicidae) or nereid (Order Phyllodocida, Family Nereididae) polychaetes (i.e., the presumed prey of *C. ebraeus* [21], [30]–[33]). We extracted DNA from single pieces of feces with the E.Z.N.A.™ Mollusc DNA Kit (Omega Bio-Tek, Doraville, Georgia, USA) as per manufacturer's recommendations. We amplified a region of the mitochondrial 16S gene with annelid-specific primers 16SANNF2 (GCGGTATCCTGACCGTGCWAAGGTA) or 16SANNF3 (GTATCCTGACCGTGCWAAGGTAGC) and 16Spr1 (CTCTAAGCCAACATCGAGGTG); the two downstream primers, 16SANNF2 and 16SANNF3, were modified from the annelid-specific primer 16SANNF (GCGGTATCCTGACCGTRCWAAGGTA) designed by Sjölin et al. [34]. In some cases, two rounds of PCR were needed to produce sufficient quantities of template for sequencing. Amplification products were diluted 1∶5 in water and used directly as sequencing templates. Sequencing was performed in both directions at the University of Michigan DNA Sequencing Core facilities. Chromatograms were examined with Sequencher 4.8 (Gene Codes Corporation, Ann Arbor, Michigan, USA). Sequences were aligned using Se-Al v2.0a11 [35] to sequences of polychaetes from GenBank. Phylograms were constructed using neighbor-joining based on Kimura 2-parameter distances with PAUP* [36].

### Comparisons of diets among populations

We compared published data on diets of *C. ebraeus* from Okinawa (reference) and Hawaii [21] to data we recovered from individuals from Guam. We used data from individuals with shell lengths ≥13 mm (i.e., adult snails) from Okinawa and combined information of prey items recovered from the alimentary tracts of adult snails from multiple locations from Hawaii. We calculated proportional similarity indices (PS_I_) [37] to estimate the levels of dietary differences at Guam and Hawaii; PS_I_ values are independent of sample size [38]. We also used G-test of independence and Fisher's exact test [39] for comparisons in which expected frequencies were <5 to test if diets differ significantly among locations.

## Results

### Conotoxin *E1* alleles

We obtained *E1* genotypes for 18 individuals of *C. ebraeus* from Hawaii, 29 individuals from Guam and 18 individuals from Okinawa (Table 1). The alleles inferred from direct sequencing of amplification products from individuals from Okinawa were identical to those inferred from cloning. Genotype information for two additional individuals from Hawaii were obtained from data reported in Duda and Palumbi [5]. While the *E1a* allele reported here is the same as that reported by Duda and Palumbi [5], their *E1b* allele corresponds to our *E1d* allele.

**Table 1:** Sample size, numbers of alleles and diversity statistics of *Conus ebraeus* conotoxin locus *E1*.

Location	Number of individuals	Number of alleles (unique^1^)	Gene diversity (SE^2^)	Nucleotide diversity (SE^2^)
Okinawa	18	9 (4)	0.837 (0.039)	0.125 (0.065)
Guam	29	6 (2)	0.682 (0.041)	0.092 (0.048)
Hawaii	20	4 (1)	0.553 (0.070)	0.088 (0.046)

1 number of unique alleles observed.

2 standard error.

**Figure 1:**
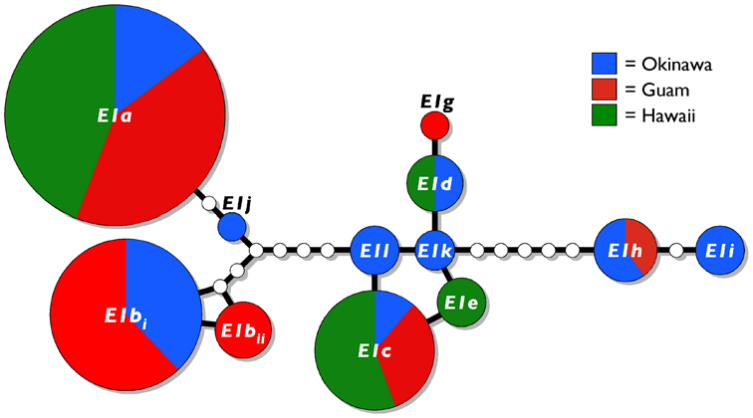
Haplotype network of alleles of conotoxin locus *E1* of *Conus ebraeus* at Guam and Hawaii. Haplotypes are illustrated as circles; hypothetical haplotypes that were not observed are illustrated as small, empty circles. Areas of circles are proportional to frequencies of alleles; pie diagrams illustrate the allelic frequencies at each location.

**Figure 2:**
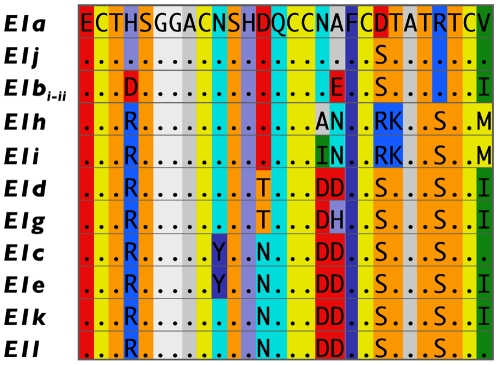
Predicted amino acid sequences of the toxin coding region of alleles of conotoxin locus *E1* of *Conus ebraeus*. Amino acids are provided as single letter codes; dots are used to indicate identity to the amino acid of the first sequence given. To illustrate radical amino acid substitutions, amino acids with similar chemical properties are provided in similar background colors (coloring based on the ‘amino’ scheme utilized in Jmol, a Java viewer for chemical structures (http://www.jmol.org/)). Amino acid sequences of *E1b_i_* and *E1b_ii_* were combined because they exhibit no nonsynonymous substitutions with the toxin coding region.

We identified 12 alleles from the 67 individuals examined (GenBank accession numbers FJ804530-FJ804536 and FJ834433-FJ834437) (Table 1). These alleles exhibit 16 polymorphic sites and differ at 1 to 11 nonsynonymous sites within the toxin coding region of the sequences that are responsible for 1 to 8 amino acid substitutions (Figs. 1, 2). Allele *E1b_ii_* differs uniquely from all other alleles at a nonsynonymous site in the prepro region of the transcript, the only substitution observed outside of the toxin coding region. Although all nucleotide substitutions are associated with amino acid substitutions, a few occur in codons that exhibit two or more changes and in these cases one of the possible substitutional pathways involves a synonymous substitution. But otherwise no synonymous substitutions were observed among alleles.

Three alleles are common and comprise 80.6% of the alleles observed in combined samples from Okinawa, Guam and Hawaii: *E1a* (45.5%), *E1b_i_* (21.6%) and *E1c* (13.4%). Two of these common alleles (*E1a* and *E1c*) occurred at all three locations (Fig. 1), but one (*E1b_i_*) was absent from Hawaii even though it was relatively frequent at both Guam (31.0%) and Okinawa (30.6%) and was either the most common allele (Okinawa) or second most common allele (Guam) at these locations. Otherwise, Hawaii has only one other allele in common with Okinawa (*E1d*), and Guam and Okinawa share just one other allele (*E1h*). The other seven alleles were unique to single locations (Table 1, Fig. 1), and these alleles were relatively rare with frequencies less than or equal to 10.0% at each location. Okinawa exhibits the greatest diversity of alleles and Hawaii exhibits the lowest (Table 1, Fig. 1). Pairwise *Φ*_ST_ values calculated from these data were large and significant for all comparisons with the pairwise comparisons between Hawaii and the other two locations yielding the largest values of *Φ*_ST_ (Table 2). Results from AMOVA showed that while most of the genetic variance was partitioned within populations (89.8%), a significant proportion of the variance was partitioned among populations (10.2%) and gave a highly significant *Φ*_ST_ value (0.102, *P*<0.00001).

**Table 2:** Pairwise *Φ*_ST_ values among populations of *Conus ebraeus* estimated from analysis of sequences of conotoxin locus *E1* (below diagonal) and mitochondrial *COI* sequence data (above diagonal).

	Okinawa	Guam	Hawaii
Okinawa		−0.007^NS^	0.022^NS^
Guam	0.069*^P^* ^ = 0.010^		−0.004^NS^
Hawaii	0.137*^P^* ^ = 0.002^	0.111*^P^* ^ = 0.0011^	

Probabilities that observed *Φ*_ST_ values deviate from a null hypothesis of no difference between populations were determined from the proportion of 10,100 permutations of haplotypes between populations that gave *Φ*_ST_ values greater than or equal to the observed *Φ*_ST_ (NS = not significant).

### *COI* haplotype data

**Figure 3:**
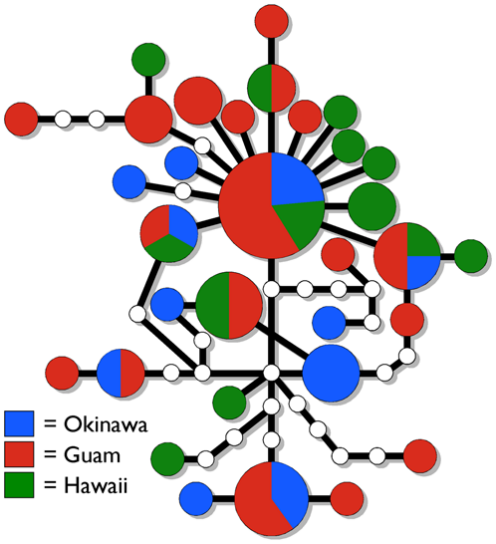
Haplotype network of *COI* sequences from 67 individuals of *Conus ebraeus* at Okinawa, Guam and Hawaii. Haplotypes are illustrated as circles, with hypothetical haplotypes that were not observed illustrated as small, empty circles. Areas of circles are proportional to the haplotype frequencies; pie diagrams illustrate frequencies of haplotypes at each location.

We obtained *COI* sequences from 22 individuals of *C. ebraeus* from Guam (GenBank accession numbers FJ804508-FJ804529) and combined these with published sequences from 10, 18 and 17 individuals from Guam, Okinawa and Hawaii respectively [18] (GenBank accession numbers provided in Methods). No obvious structure is apparent in the network constructed from the haplotype sequences (Fig. 3). Moreover, in contrast to results from analysis of conotoxin *E1* alleles, the pairwise *Φ*_ST_ values are small and not significantly different from zero (Table 2). Also, results from AMOVA showed that only a small proportion of the variance was partitioned among populations (0.03%) and gave a small and insignificant *Φ*_ST_ value (0.0003, *P* = 0.430).

### Dietary analyses

**Figure 4:**
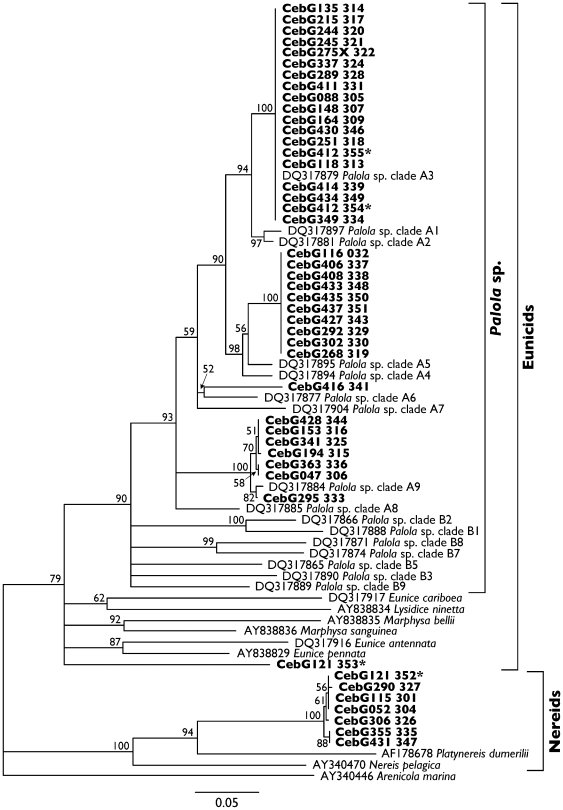
Neighbor-joining tree constructed from Kimura 2-parameter distances [48] of sequences of a region of the mitochondrial 16S gene. Bootstrap values from 1000 replicates are presented on branches; branches with bootstrap values less than 50% were collapsed. The tree is rooted to a sequence from *Arenicola marina* (Order Capitellida, Family Arenicolidae). Sequences obtained from feces are indicated in bold; names include the specimen code and fecal sample number. Names of sequences of polychaetes from GenBank are given with their GenBank accession numbers. Sequences from fecal materials of CebG121 and CebG412, the two individuals that defecated twice over a 24-hour period, are indicated with asterisks.

We examined 52 fecal samples of 50 individuals of *C. ebraeus* from Guam. We recovered putative 16S sequences from 50 of the 52 samples, but 5 of these sequences did not align well with 16S sequences of annelids. Four of these sequences were similar to published sequences of ribosomal RNA genes of bacteria, including *Bacillus cereus* (*n* = 3) and *Frankia* (*n* = 1). Based on results from NCBI BLASTn searches, the other sequence showed similarity to rRNA sequences of various protists and bacteria, but exhibited poor matches to any particular sequences in GenBank. Because fecal samples of these five samples contained obvious hard parts of eunicid or nereid polychaetes, we suspect that DNAs from prey were highly degraded and our primers amplified templates of other species that were present in the feces or collected along with the fecal materials. The other 45 sequences (GenBank accession numbers FJ804537-FJ804572 and FJ907334-FJ907342) aligned well with either eunicid or nereid polychaete 16S sequences, including 37 sequences that exactly matched or were very similar to published sequences of *Palola* sp. [40] (Fig. 4). Most sequences (*n* = 19) were identical to sequences of Schulze's [40] *Palola* clade A3 that were detected by Schulze at several locations in the western Pacific (e.g., Guam, Pohnpei, Yap) (Fig. 4). The second most common set of 16S haplotypes (*n* = 10) group with sequences of *Palola* (clades A4 and A5) but they did not match any *Palola* sequences reported by Schulze [40] (Fig. 4). Seven other sequences group with Schulze's [40] sequences of *Palola* clade A9 from Micronesia (Kosrae and Pohnpei) (Fig. 4). Seven of the eight sequences that did not cluster with sequences of *Palola* in phylogenetic reconstructions exhibited few (0–3) nucleotide substitutions amongst each other and clustered with sequences of various nereids; the eighth sequence grouped with eunicids but was clearly not *Palola* (Fig. 4). Of the two individuals that defecated twice, one apparently consumed both a nereid and an unidentified eunicid (CebG121, see Fig. 4). Sequences from the other were identical to sequences from *Palola* clade A3 reported by Schulze [40]; either the individual consumed two *Palola* worms or feces from the same prey item were defecated twice. Based on microscopic examination, 43 fecal samples contained structures that resembled features of eunicid polychaetes; 9 others contained structures characteristic of nereids. These identifications based on polychaete hard part anatomy were entirely consistent with the DNA-based identifications.

*C. ebraeus* from Hawaii shows differences in the proportions of eunicids and nereids that are consumed in comparison to individuals from Okinawa and Guam (Table 3) and respective similarity indices are low (Hawaii-Guam: PS_I_ = 0.572; Hawaii-Okinawa: PS_I_ = 0.483). These differences are highly significant for both comparisons G>17 and *P*<0.0001. On the other hand, eunicids (i.e., *Palola* sp.) and nereids comprise a nearly equivalent proportion of the diets of snails at Guam and Okinawa (PS_I_ = 0.911) and diets are not significantly different (G = 0.873, *P* = 0.35; Fisher's exact test: *P* = 0.67). We conducted all tests based on the frequencies of combined eunicid and nereid polychaetes consumed at each location and did not include frequencies of other polychaete families.

**Table 3:** Prey recovered from gut contents or feces of *Conus ebraeus* from locations in the Indo-West Pacific.

	Okinawa [20]	Guam	Hawaii [21]
Nereididae			
* Perinereis helleri*			143
Unidentified nereids	1	7	
Total nereids	1	7	143
Eunicidae			
* Palola* sp.	14	37	95
* Eunice cariboea*			2
* Lysidice collaris*			4
* Unideintified eunicids*		1	1
* Total eunicids*	14	38	102
Other families or unidentified annelids	2		3

## Discussion

Our analyses reveal significant differences in allelic frequencies of conotoxin locus *E1* of *C. ebraeus* from Okinawa, Guam and Hawaii (Table 2). On the contrary, results from examination of additional *COI* sequences from Guam corroborate inferences by Duda and Lessios [18] that *C. ebraeus* exhibits no genetic differentiation among locations in the western and central Pacific (Table 2). Analysis of fecal samples shows that *C. ebraeus* at Guam predominantly preys on eunicid polychaetes (*Palola* sp.) (Table 3; Fig. 4). This latter result is consistent with observations of diets of *C. ebraeus* at Okinawa [20] as well as at the western Indian Ocean, the eastern Indian Ocean, the Great Barrier Reef and Papua New Guinea in the Indo-West Pacific [30]–[33], but contrasts with data from Hawaii [21] as well as the Seychelles [41] where nereid polychaetes comprise a larger proportion of prey items (see Table 3).

### Geographic variation of conotoxin *E1* allelic frequencies

The significant differences in allelic frequencies of an expressed conotoxin locus at Okinawa, Guam and Hawaii contrasts strongly with the lack of genetic differentiation observed at *COI* sequences from these locations (Table 2; Figs. 1, 3). Mitochondrial gene sequences tend to coalesce four times faster than nuclear genes due to the uniparental inheritance and haploid condition of the mitochondrial genome [42]. Thus the significant differences in allelic frequencies at conotoxin locus *E1* are unlikely to be solely due to neutral mechanisms (i.e., drift) and so localized selection pressures may have contributed to the genetic differentiation of this locus at these locations. As implied by the strong segregation of *E1* alleles in light of the apparent lack of genetic structure observed at mitochondrial gene sequences, selection may be strong enough to counteract the homogenizing effect of gene flow. Alternatively, *C. ebraeus* may have experienced a recent selective sweep of mitochondrial genomes, but this seems unlikely given the diversity of haplotypes observed (Fig. 3).

We have not examined the functions of the predicted peptide sequences of the *E1* alleles, but conotoxin peptides that differ at single amino acid positions show differences in function [43], [44]. Moreover, many of the amino acid substitutions result in charge differences among the translated peptides (Fig. 2) and all nucleotide substitutions among alleles are associated with amino acid substitutions. Thus, the sequence variation exhibited by the *E1* alleles is presumably manifested as functional variation in the gene products of these alleles. Aside from alleles *E1h* and *E1i*, most of the amino acid variation is exhibited by the three most commonly observed alleles (*E1a*, *E1b_i_* and *E1c*) and these show six to ten nucleotide substitutions between them that are responsible for four to seven amino acid differences in the translated peptides. One of the common alleles (*E1b_i_*) is absent at Hawaii. On the other hand, most of the rare alleles (*E1b_ii_*, *E1d*, *E1e*, *E1g*, *E1j*, *E1k* and *E1l*) are just a few nucleotide substitutions removed from the three common alleles. Thus, much of the presumed functional diversity of the gene products of the alleles of locus *E1* is unequally distributed among these locations. As a whole, Okinawa apparently exhibits the greatest functional diversity at this locus.

### Geographic variation in diets of *Conus ebraeus*

While the focal prey of *C. ebraeus* are eunicid polychaetes (i.e., members of the genus *Palola*) at most locations in the Indo-West Pacific (e.g., the Maldives, eastern Indian Ocean, Great Barrier Reef, Okinawa and Guam) [30]–[33], at Hawaii and the Seychelles this species predominantly preys on nereid polychaetes [21], [41]. These results suggest that *C. ebraeus* exhibits geographic variation in dietary specialization and the diet of this species is distinct at Hawaii and the Seychelles compared to at other locations. The distinctiveness of the diets at Hawaii and the Seychelles could though be explained by seasonal variation or ontogenetic shifts in diets of *C. ebraeus*, and microhabitat differences in prey availability. Diets of *Conus* populations are relatively fixed and no major differences in diets of populations have been observed over short time scales, during different times of the year or across short microgeographic scales (A.J. Kohn, University of Washington, personal communication). Thus, we suspect that seasonal changes in diet are unlikely to explain the unique diet observed at Hawaii and Seychelles, especially given the large number of specimens that were examined and extent of the sampling at Hawaii [21]. At Okinawa, *C. ebraeus* shows definite changes in diet during development: juvenile and subadult individuals (shell lengths <13 mm) prey solely on syllid polychaetes (Order Phyllodocida, Family Syllidae) and adults (shell lengths ≥13 mm) predominantly eat eunicid polychaetes [20]. Nonetheless, we restricted our comparisons to diets of adult snails and so this factor seems unlikely to be responsible for the observed unique diets of *C. ebraeus* at Hawaii and the Seychelles. Kohn [21] studied diets of *C. ebraeus* from a variety of locations around the island of Oahu, Hawaii. These locations included several marine bench habitats and several subtidal reef habitats, the latter of which are probably similar in characteristics to the locations where we studied *C. ebraeus* from Guam and where Duda *et al.* [20] studied it at Okinawa. Kohn [21] observed that *C. ebraeus* diets differ among these habitats, presumably reflecting differences in prey availability, with nereid polychaetes comprising a greater proportion of prey items at marine benches than at subtidal reef habitats. Nereids may be more common within the algal mats of reef benches than in subtidal reef habitats (A.J. Kohn, University of Washington, personal communication). But at the Seychelles, *C. ebraeus* occurred predominantly in microhabitats with few algae and so the preference for nereids at this location cannot be explained by differences in the availability of prey associated with particular microhabitats. Therefore, although we cannot necessarily exclude the possibility that the observed differences in diet of *C. ebraeus* stem from variation in prey abundance among different microhabitats where this species occurs (especially at Hawaii), it seems unlikely given the data from the Seychelles.

### Sources of selection

Because *Conus* venoms are used primarily to subdue prey, selection at *E1* may be associated with geographic differences in dietary specialization, possibly at Hawaii given the greater proportion of nereid polychaetes consumed by *C. ebraeus* here (but see above). Thompson's [45] coevolutionary alternation with escalation hypothesis posits that predators' specializations for prey alternate and these changes could result in the rapid evolution of characters related to predation in predators. Accordingly, the evolution of *Conus* venoms may be strongly affected by dietary shifts such that changes in venom composition are driven by changes in specializations for particular prey, a process that presumably occurred in *C. miliaris* at Easter Island [15]. In particular, differences in the availability of prey between locations may lead to location-specific dietary specializations and localized selection pressures on venoms. But this proposition cannot explain the differences in allelic frequencies at Guam and Okinawa where diets of *C. ebraeus* are nearly identical (Table 3). Instead, *C. ebraeus* may exhibit geographic variation in the intensity or direction of selection pressures that emanate from predator-prey interactions such that coevolution of *C. ebraeus* and its prey exhibit a mosaic pattern of coevolutionary hotspots and coldspots *sensu* Thompson's (2005) geographic mosaic theory of coevolution. Clearly results from additional investigations are needed to test these hypotheses.

### DNA-based dietary analyses of *Conus*

Although DNA-based methods for identifying diets of various taxa were developed some time ago [46], [47], to our knowledge this is the first time this approach has been utilized for examining diets of *Conus*. Our current success rate is reasonably high (i.e., roughly 87% of the fecal samples yielded polychaete sequences); improved extraction techniques or different amplification methods could improve this rate. More importantly, the level of resolution in identifying diets of *Conus* offered from recovery of prey 16S sequences is clearly superior to that provided by traditional morphological-based identification of *Conus* prey. For example, while DNA-based methods show that prey of *C. ebraeus* at Guam are members of four divergent clades of *Palola* sp. (Fig. 4), morphological-based methods would not be able to determine this because the members of these clades exhibit no morphological synapomorphies [40].

### Conclusions

We found that *C. ebraeus* exhibits significant differences in conotoxin allelic frequencies at Okinawa, Guam and Hawaii despite results from analysis of mitochondrial *COI* sequences that suggest that no population genetic structure occurs among these locations. These patterns imply that selection is responsible for the differences in allelic frequencies of the conotoxin locus and that neutral mechanisms alone cannot explain these observations. Although presumed differences in diet at Hawaii could be associated with differences in allelic frequencies at this location, *C. ebraeus* at Okinawa and Guam exhibit nearly identical diets and yet allelic frequencies differ significantly between these locations as well. Thus, divergence in allelic frequencies may result from differences in selection pressures not associated with differences in dietary specialization, such as variation in the intensity of predator-prey arms races at different locations. Presumably similar factors generate intraspecific variation in venom composition and the geographic segregation of this variation in other venomous organisms, but additional studies of geographic variation in venom composition and its functional significance are clearly needed to more thoroughly evaluate these assertions.
